# Supplementary material for: Toll like Receptor signalling by Prevotella histicola activates alternative NF-κB signalling in Cystic Fibrosis bronchial epithelial cells compared to P. aeruginosa
Source: PLoS One. 2020 Oct 8;15(10):e0235803. doi: 10.1371/journal.pone.0235803 (PMC7544055; doi:10.1371/journal.pone.0235803)
Supplement: S1 Text — (DOCX) [file pone.0235803.s001.docx]

Toll like Receptor signalling by Prevotella histicola activates alternative NF-κB signalling in Cystic Fibrosis bronchial epithelial cells compared to *P. aeruginosa*

**A. Bertelsen ^a,b^, J.S. Elborn ^a,c^, B.C. Schock ^a,^***

**Supporting information – Materials & Methods**

**Clinical isolates:**

The clinical isolate of *P. histicola* was grown under anaerobic conditions using a Don Whitley anaerobic cabinet (Don Whitley A35 workstation) on Columbia Blood Agar (CBA, Fannin LIP) for 48 hours. This lawn of colonies was used to inoculate 10mL of anaerobic basal broth (Oxoid)TO OD.0.1 and this was allowed to grow to mid log phase (approximately 18 hours). This culture was used for infection experiments. A clinical isolate of *P. aeruginosa* was grown under standard aerobic conditions on CBA over-night and used to inoculate 10 mL LB broth. Start OD was 0.05. This broth culture was grown until mid-log phase under aerobic conditions (approximately 3-4 hours) and used for infection experiments.

**Bacterial growth under anaerobic conditions:**

To ensure that differences in inflammatory signalling during infection of CFBE41o- cells were not due to different bacterial growth, the clinical isolates *P. histicola* B011L and *P. aeruginosa* B021 were cultured under anaerobic conditions for 4 hours in antibiotic free MEM medium using a Don Whitley anaerobic cabinet as described (1). Comparison revealed no differences in growth rates between the two species (S 1 Fig).

**S1 Fig: Growth rate of *P. histicola* and *P. aeruginosa*.**

**Infection assays:**

CFBE41o- cells were infected with *P. histicola* or *P. aeruginosa* at an MOI (Multiplicity of Infection) of 100 for 4 hours. Both bacteria were grown to mid log phase as described previously (2). An MOI of 100 was defined by plating all inocula on CBA agar and enumerating viable counts the following morning. Liquid cultures were used to inoculate cells for infection experiments and cells were incubated for up to 4 hours under anaerobic conditions as described above. Cells were incubated under anaerobic conditions for the duration of the experiments as described in supplementary data.

**Cell viability assays:**

1. *LDH assay:* Cell death was assessed by measuring LDH release from infected cells and non-infected control cells. 10μL of supernatant was used for each assay as per manufacturers’ instructions (Abcam, ab69693).
2. *MTT assay:* The measurement of mitochondrial activation is used as a surrogate for cell viability. In brief, NADPH dependent mitochondrial enzymes convert yellow MTT (3- [4, 5-dimethyl thiazol-2yl] – 2, 5 diphenyl tetrazolium bromide) to purple formazan crystals. MTT (stock solution 5mg/mL) was used at a final concentration of 15µg/mL MTT was added to each well and cells were incubated for 4 hours at 37°C. The resulting crystals were dissolved in solubilisation buffer (HCL-SDS, 600 µL/well (6-well plate)) and cells were incubated at 37°C with 5% CO_2_ for an additional four hours. Samples were homogenised transferred into a 96-well plate and absorbance at λ=570nm determined.
3. *Trypan Blue exclusion assay:* Trypan blue (Sigma) was sterile filtered and equal volumes (usually 10μl) of cell suspension and dye were mixed in Eppendorf reaction tubes and immediately loaded onto a Neubauer haemocytometer. Cells which appeared blue under the microscope were determined as ‘dead’ and cells appearing white were counted as ‘live cells’.

To advise incubation conditions for the anaerobic experiments with CFBE41o- cells, cells were incubated aerobically (antibiotic free MEM, 37°C, 5% CO_2_, 95% mixed gas) and anaerobically (antibiotic free MEM, 10% FBS, 37^o^C, 10% CO_2,_ 10% H_2_, 80% N_2_) for up to six hours. MTT activity, LDH releases and Trypan Blue exclusion coherently showed a significant increase in cell death at 6-hour anaerobic incubation (S2 Fig). Based on these results experiments with CFBE41o- cells were only conducted for a maximum of four hours in the anaerobic cabinet.

**S2 Fig: Cell viability under aerobic and anaerobic conditions.**

**TLR Reporter Assays:**HEK-293-TLR2, HEK-293-TLR4 and HEK-293-TLR5 cells were purchased from InvivoGen and maintained in high Glucose DMEM with 10% FBS, L-Glutamine and Pen/Strep as per manufacturer’s instructions. 100μg Blasticidin was added to cells after the second passage as per manufacturer’s instructions and maintained in the media thereafter. Transfection of cells was carried out as per manufacturer’s instructions using LyoVec and pNifty-Luc and incubated for 24 hours under standard tissue culture conditions to recover from the transfection. Infection of the cells was carried out as described previously and cells were incubated under anaerobic conditions for the duration of the experiments

**Cytoplasmic and nuclear fraction extraction for DNA binding ELISA:**

Cells were infected as described previously. Cytoplasmic and nuclear fractions were extracted from cell populations either as directed by the kit manual (IKKα ELISA) or using the NuPer nuc,ear extraction kit (Thermo Fisher). Total protein was quantified by BCA assay (Thermo Fisher) and 20μg nuclear fraction and 100μg total cytoplasmic fraction were used for subsequent transcription factor ELISA.

**Innate Immune Response Profile:**Antibacterial innate immune response profilers were purchased from SaBiosciences. Cells were infected as described previously. Total RNA was extracted using a combination of Trizol and column purification. 500ng total RNA was reverse transcribed to cDNA using the Qiagen Quantitecht cDNA synthesis kit as recommended by the manufacturer. Genomic DNA was eliminated from all samples using the Qiagen genomic DNA Wipeout buffer as per manufacturer’s instructions. cDNA was used for all RT profiler assays.

**Gene (mRNA) expression analyses:**

Trizol was used for cell lysis and a combination of chloroform phenol and column extraction was used to extract total RNA from control and infected cells. Trizol was used at a 1mL volume/1*10^6^ cells. Chloroform was used at a volume of 200μL / 1*10^6^ cells. After the addition of chloroform to each sample, samples were incubated for 5 minutes at room temperature and were subsequently centrifuged at 12000xg at 4° C for 15 minutes. The aqueous phase containing the RNA was removed, an equal volume of 70 % molecular grade EtOH was added to each sample and each sample was loaded onto an RNA extraction column to purify the RNA (Thermo-Fisher, 12183018A).

Samples were purified as per manufacturer’s instructions and total RNA was quantified using the Nano-drop 2000. 500ng of total RNA was reverse transcribed using the SuperScript III reverse transcription kit (Thermo Fisher 18080044). DNA removal was carried out on the column using Turbo DNase (AM2238) from Thermo-Fisher.Quantitative PCR was carried out for a total of 40 cycles on an MX300-P (Agilent technologies) using a fast SybR Green master-mix as per manufacturer’s instructions (Thermo Fisher 4385612) with Qiagen gene specific primers (Qiagen QT00000323, QT00083720, QT00199752). Controls included no RT controls, water controls and master-mix controls for each primer. Melt-curve analysis was carried out for each sample to ensure no contamination of the samples and to further ensure that primer dimers were not created during the cycling process. Data was assessed using the ΔΔCT method of analysis.

**siRNA assays and transfection:**

Lipofectamine 2000 (Thermo Fisher Scientific, 11668019) was used in all knock-down experiments as previously described (3). Silencer select siRNAs was purchased from Thermo Fisher Scientific for TLR2 (s169), TLR5 (s14197) and IKKα (s3078) and scrambled siRNA (4390843). An AllStars siRNA positive control was used to validate all assays (Qiagen, SI03650318). All purchased siRNA sequences were reconstituted to a concentration of 25 μM in nuclease free water (Thermo Fisher, AM9935). Serum free Opti-MEM was also used for all transfection assays (Thermo Fisher Scientific, 31985070).

Assays examining the effect of Lipofectamine 200 on the cells (sham transfection), the effect of the scrambled siRNA , the efficacy of transfection and off target effects using bacteria and scrambled siRNA showed that 20 pmol/mL of each siRNA in a 1:20 dilution of Lipofectamine 2000: OptMem was sufficient to induce 75%-80% target knockdown in CFBE41o- cells at 24 hours post transfection for gene expression, with corresponding protein expression affected by 48 hours post transfection. All Stars siRNA was used as a positive control in all experiments. To ensure cell viability after transfection, cell viability was determined in sham transfected cells. Lipofectamine did not induce any significant increase in LDH release (S3 Fig).

**S3 Fig: Cell viability during transfection.**

The specificity of siRNA target knock-down for TLR5 and subsequent IL-6 release after the infections (*P. aeruginosa, P. histicola*) or stimulation with flagellin was also investigated. SiRNA at 20 pmol/mL in a 1:20 dilution of Lipofectamine 2000:Opti-Mem was sufficient to induce a significant reduction in TLR5 (55% and 60% reduction in TLR5 mRNA in response to *P. histicola* and *P. aeruginosa* infection, respectively) (S4 Fig, A), followed by a ≥70% reduction IL-6 mRNA (48 hours post transfection) (S4 Fig, B), similar to the conditions described previously (2)). Positive control AllStars siRNA (Qiagen, SI03650318) was used in all experiments.

**S4 Fig: Transfection efficiency for target genes.**

**References**

1. Tunney MM, Field TR, Moriarty TF, Patrick S, Doering G, Muhlebach MS, Wolfgang MC, Boucher R, Gilpin DF, McDowell A, Elborn JS. 2008. Detection of anaerobic bacteria in high numbers in sputum from patients with cystic fibrosis. Am J Respir Crit Care Med 177:995-1001.

2. Bertelsen A, Elborn JS, Schock BC. 2019. Infection with Prevotella nigrescens induces TLR2 signalling and low levels of p65 mediated inflammation in Cystic Fibrosis bronchial epithelial cells. Journal of Cystic Fibrosis.

3. Gilpin DF, Nixon KA, Bull M, McGrath SJ, Sherrard L, Rolain JM, Mahenthiralingam E, Elborn JS, Tunney MM. 2017. Evidence of persistence of Prevotella spp. in the cystic fibrosis lung. J Med Microbiol doi:10.1099/jmm.0.000500:825-832.
