# Supplementary figures and images for: Toll like Receptor signalling by Prevotella histicola activates alternative NF-κB signalling in Cystic Fibrosis bronchial epithelial cells compared to P. aeruginosa
Source: PLoS One. 2020 Oct 8;15(10):e0235803. doi: 10.1371/journal.pone.0235803 (PMC7544055; doi:10.1371/journal.pone.0235803)

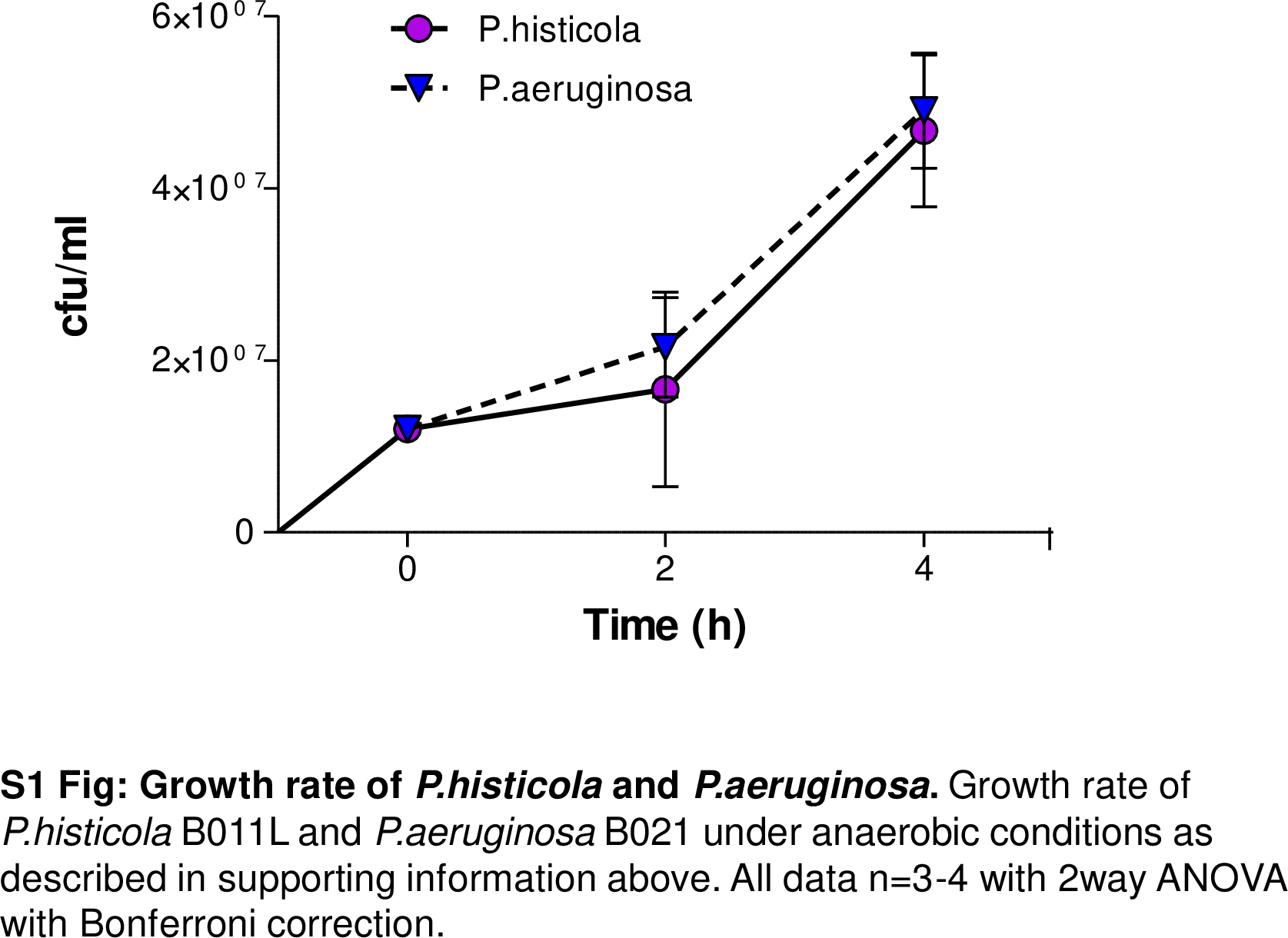

Supplement: S1 Fig — Growth rate of P. histicola B011L and P. aeruginosa B021 under anaerobic conditions as described in supporting information above. All data n = 3–4 with 2way ANOVA with Bonferroni correction. (TIF) [file pone.0235803.s002.tif]

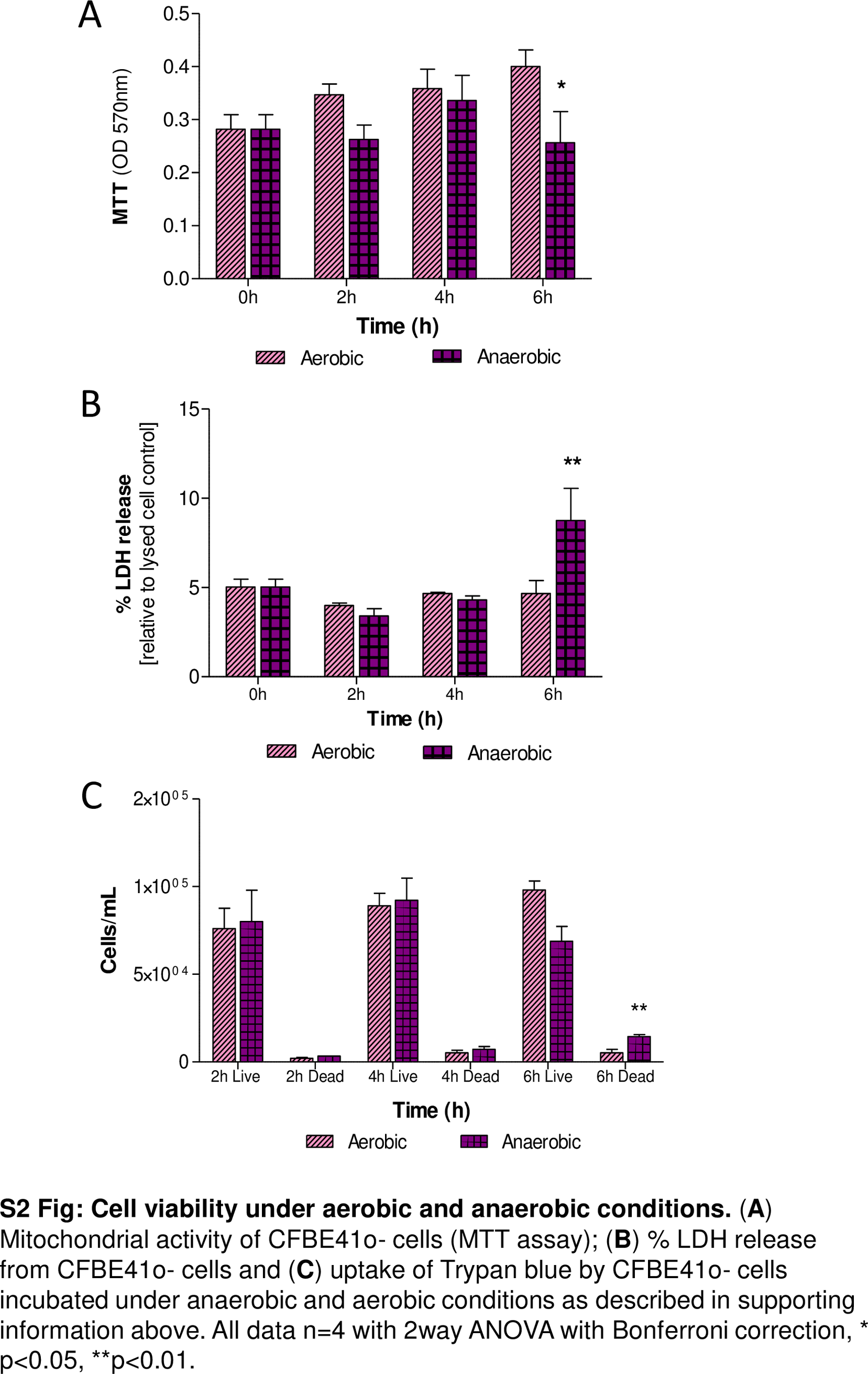

Supplement: S2 Fig — (A) Mitochondrial activity of CFBE41o- cells (MTT assay); (B) % LDH release from CFBE41o- cells and (C) uptake of Trypan blue by CFBE41o- cells incubated under anaerobic and aerobic conditions as described in supporting information above. All data n = 4 with 2way ANOVA with Bonferroni correction, *p<0.05, **p<0.01. (TIF) [file pone.0235803.s003.tif]

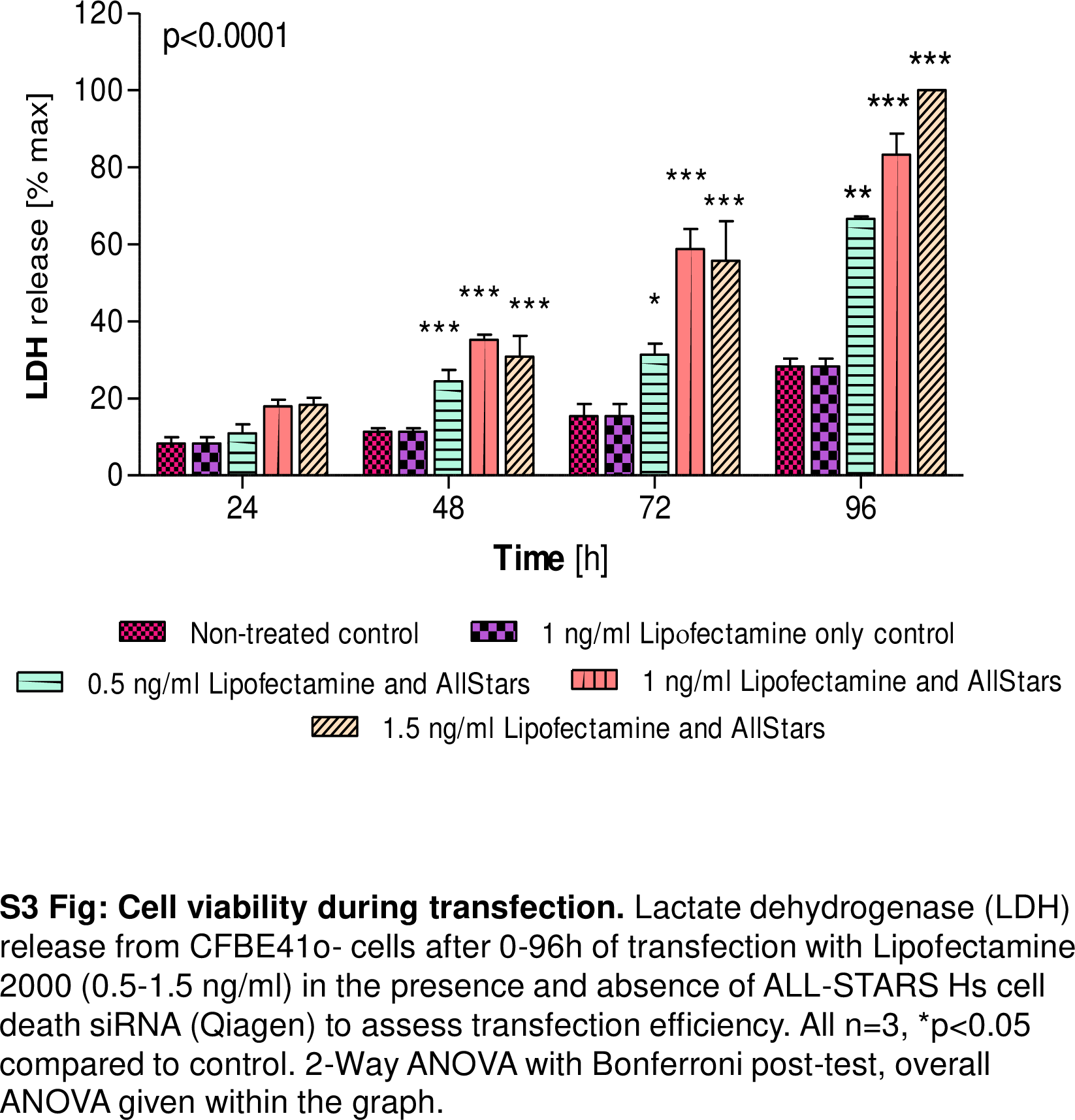

Supplement: S3 Fig — Lactate dehydrogenase (LDH) release from CFBE41o- cells after 0-96h of transfection with Lipofectamine 2000 (0.5–1.5 ng/ml) in the presence and absence of ALL-STARS Hs cell death siRNA (Qiagen) to assess transfection efficiency. All n = 3, *p<0.05 compared to control. 2-Way ANOVA with Bonferroni post-test, overall ANOVA given within the graph. (TIF) [file pone.0235803.s004.tif]

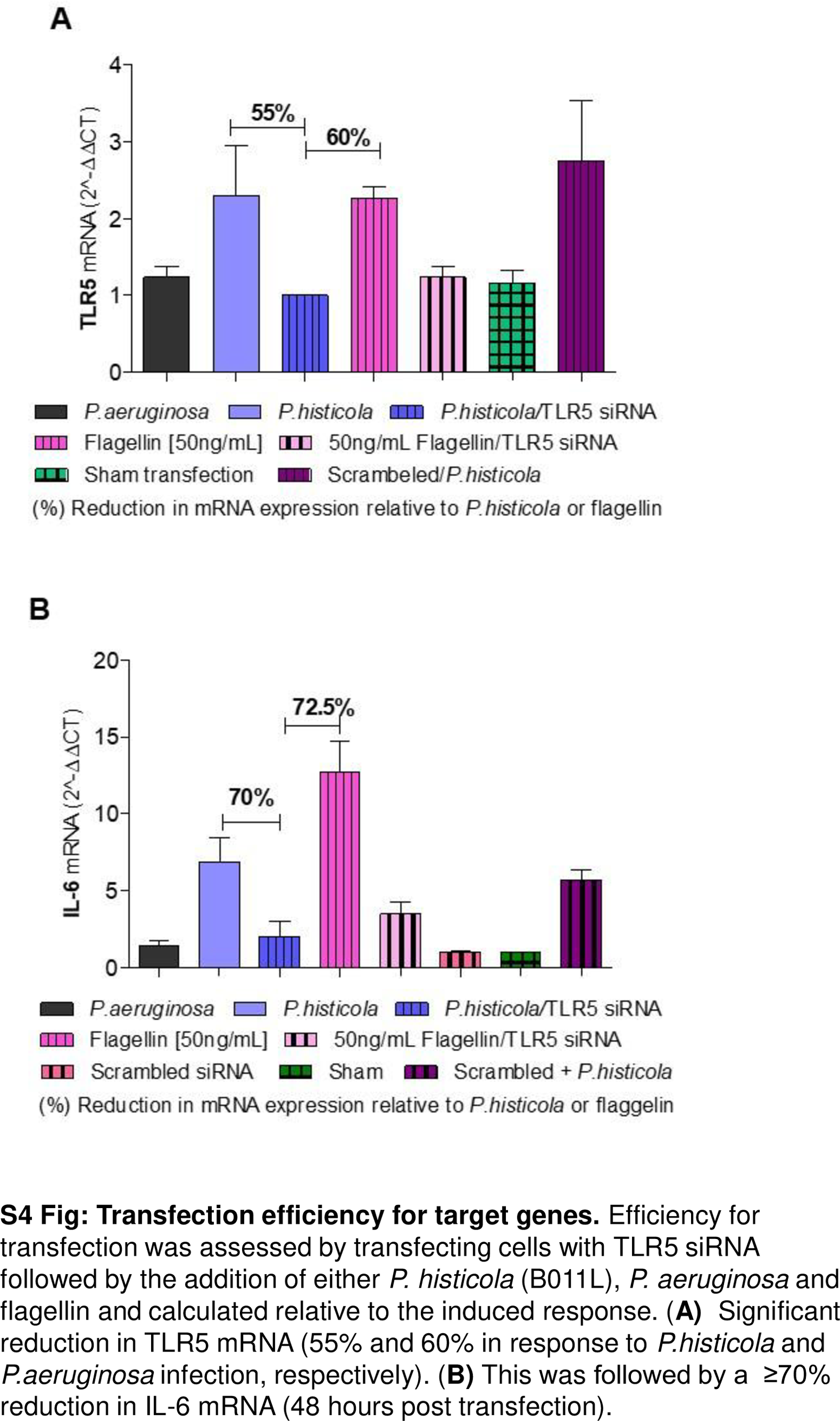

Supplement: S4 Fig — Efficiency for transfection was assessed by transfecting cells with TLR5 siRNA followed by the addition of either P. histicola (B011L), P. aeruginosa and flagellin and calculated relative to the induced response. (A) Significant reduction in TLR5 mRNA (55% and 60% in response to P. histicola and P. aeruginosa infection, respectively). (B) This was followed by a ≥70% reduction in IL-6 mRNA (48 hours post transfection). (TIF) [file pone.0235803.s005.tif]
